# PINK1 Protects against Oxidative Stress by Phosphorylating Mitochondrial Chaperone TRAP1

**DOI:** 10.1371/journal.pbio.0050172

**Published:** 2007-06-19

**Authors:** Julia W Pridgeon, James A Olzmann, Lih-Shen Chin, Lian Li

**Affiliations:** Department of Pharmacology, Emory University School of Medicine, Atlanta, Georgia, United States of America; Baylor College of Medicine, United States of America

## Abstract

Mutations in the *PTEN induced putative kinase 1 (PINK1)* gene cause an autosomal recessive form of Parkinson disease (PD). So far, no substrates of PINK1 have been reported, and the mechanism by which PINK1 mutations lead to neurodegeneration is unknown. Here we report the identification of TNF receptor-associated protein 1 (TRAP1), a mitochondrial molecular chaperone also known as heat shock protein 75 (Hsp75), as a cellular substrate for PINK1 kinase. PINK1 binds and colocalizes with TRAP1 in the mitochondria and phosphorylates TRAP1 both in vitro and in vivo. We show that PINK1 protects against oxidative-stress-induced cell death by suppressing cytochrome c release from mitochondria, and this protective action of PINK1 depends on its kinase activity to phosphorylate TRAP1. Moreover, we find that the ability of PINK1 to promote TRAP1 phosphorylation and cell survival is impaired by PD-linked PINK1 G309D, L347P, and W437X mutations. Our findings suggest a novel pathway by which PINK1 phosphorylates downstream effector TRAP1 to prevent oxidative-stress-induced apoptosis and implicate the dysregulation of this mitochondrial pathway in PD pathogenesis.

## Author Summary

Parkinson disease (PD) is characterized by the selective loss of midbrain dopaminergic neurons. Although the cause of PD is unknown, pathological analyses have suggested the involvement of oxidative stress and mitochondrial dysfunction. Recently, an inherited form of early-onset PD has been linked to mutations in both copies of the gene encoding the mitochondrial protein PINK1. Furthermore, increasing evidence indicates that single-copy mutations in PINK1 are a significant risk factor in the development of later-onset PD. Here we show that PINK1 is a protein kinase that phosphorylates the mitochondrial molecular chaperone TRAP1 to promote cell survival. We find that PINK1 normally protects against oxidative-stress-induced cell death by suppressing cytochrome c release from mitochondria. The PINK1 mutations linked to PD impair the ability of PINK1 to phosphorylate TRAP1 and promote cell survival. Our findings reveal a novel anti-apoptotic signaling pathway that is disrupted by mutations in PINK1. We suggest that this pathway has a role in PD pathogenesis and may be a target for therapeutic intervention.

## Abstract 3

Mutations in the gene that codes for PINK1 cause a common form of Parkinson disease. Here the authors show that PINK1 phosphorylates TRAP1, which suppresses apoptotic release of cytochrome c from mitochondria.

## Introduction

Parkinson disease (PD) is the second most common neurodegenerative disease, characterized by the selective loss of dopaminergic neurons in the substantia nigra [1]. The cause of PD, particularly the sporadic disease, is unclear, but it likely involves both genetic and environmental factors. Genetic studies have identified a number of genes associated with familial PD [2]. Postmortem analyses reveal a deficiency in the mitochondrial complex I function in patients with sporadic PD [3]. Furthermore, exposure to environmental toxins that inhibit the mitochondrial complex I can lead to PD-like phenotypes in animal models [4], suggesting the involvement of mitochondrial dysfunction in PD pathogenesis.

Mutations in the *PTEN induced putative kinase 1 (PINK1)* gene were originally discovered in three pedigrees with recessively inherited PD. Two homozygous *PINK1* mutations were initially identified: a truncating nonsense mutation (W437X) and a G309D missense mutation [5]. Subsequently, multiple additional types of PD-linked mutations or truncations in *PINK1* have been reported, making *PINK1* the second most common causative gene of recessive PD [6,7]. Interestingly, despite autosomal recessive transmission of *PINK1*-linked early-onset PD, a number of heterozygous mutations affecting only one *PINK1* allele have been associated with late-onset PD [6–10]. The pathogenic mechanisms by which *PINK1* mutations lead to neurodegeneration are unknown.

*PINK1* encodes a 581-amino-acid protein with a predicted N-terminal mitochondrial targeting sequence and a conserved serine/threonine kinase domain [5]. PINK1 protein has been shown to localize in the mitochondria [5,11–13] and exhibit autophosphorylation activity in vitro [11,12,14]. The in vivo substrate(s) and biochemical function of PINK1 remain unknown. In cultured mammalian cells, overexpression of wild-type PINK1 protects cells against apoptotic stimuli [5,15], whereas small interfering RNA (siRNA)–mediated depletion of PINK1 increases the susceptibility to apoptotic cell death [16]. In *Drosophila,* loss of PINK1 leads to mitochondrial defects and degeneration of muscle and dopaminergic neurons [17–20]. Despite ample evidence indicating an essential role of PINK1 in cytoprotection, the mechanism by which PINK1 protects against apoptosis is not understood.

Here, we describe the characterization of mitochondrial serine/threonine kinase PINK1 and report the identification of TNF receptor-associated protein 1 (TRAP1), a mitochondrial molecular chaperone also known as heat shock protein 75 (Hsp75), as a PINK1 substrate. Our results suggest that PINK1 protects against oxidative-stress-induced apoptosis by phosphorylating downstream effector TRAP1, and provide novel insights into the pathogenic mechanisms of PINK1 mutations in causing PD.

## Results

### PINK1 Interacts with TRAP1

**Figure 1:**
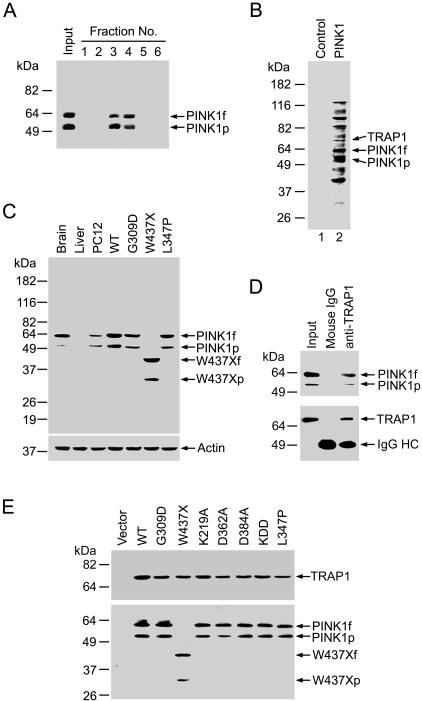
Interaction of TRAP1 with Wild-Type and Mutant PINK1 (A) Lysates of HeLa cells expressing C-terminally FLAG-tagged wild-type PINK1 (Input) were affinity purified with anti-FLAG M2 affinity gel, and the eluate fractions were analyzed by immunoblotting with anti-FLAG antibody. (B) Affinity-purified proteins from PINK1-transfected cells and vector control were resolved on SDS-PAGE and detected by Ponceau S staining. Arrows indicate three bands identified by mass spectrometry as TRAP1, PINK1f, and PINK1p. (C) Specificity of the polyclonal anti-PINK1 antibody. Homogenates (50 μg of protein per lane) from rat brain, liver, untransfected PC12 cells, and transfected PC12 cells expressing wild-type (WT) or mutant PINK1 were analyzed by immunoblotting with anti-PINK1 and anti-actin antibodies. (D) Endogenous PINK1 interacts with TRAP1. PC12 cell lysates (Input) were immunoprecipitated with anti-TRAP1 antibody, followed by immunoblotting with anti-PINK1 and anti-TRAP1 antibodies. IgG HC, IgG heavy chain. (E) Lysates from PC12 cells transfected with FLAG-tagged wild-type or mutant PINK1 were immunoprecipitated with anti-FLAG antibody, followed by immunoblotting using anti-TRAP1 and anti-FLAG antibodies.

To identify PINK1-interacting proteins, we performed affinity purification experiments using anti-FLAG M2 affinity gel to isolate proteins associated with C-terminally FLAG-tagged human wild-type PINK1 from transfected HeLa cell extracts. Consistent with previous reports [11,12], Western blot analysis detected a 63-kDa full-length form (PINK1f) and a 52-kDa processed form (PINK1p) of PINK1 protein in the cell extracts (Figure 1A*,* “Input”). Both forms of PINK1 were eluted from the affinity column in fractions 3 and 4 (Figure 1A). Ponceau S staining of the affinity-purified PINK1 immune complexes revealed the presence of several proteins that were specifically associated with PINK1 (Figure 1B). Mass spectrometry analysis unambiguously identified the 75-kDa PINK1-associated protein as TRAP1, a ubiquitously expressed protein with significant sequence homology to the HSP90AA1 family of molecular chaperones [21–23].

In order to characterize endogenous PINK1 protein and its interaction with TRAP1, we generated a rabbit polyclonal anti-PINK1 antibody against amino acid residues 135–155 of human PINK1. Western blot analysis demonstrated that the anti-PINK1 antibody, but not the preimmune serum, specifically recognized endogenous PINK1 as well as recombinant wild-type and mutant PINK1 proteins (Figure 1C; additional data not shown). Using this anti-PINK1 antibody, we found that both the full-length and the processed forms of endogenous PINK1 co-immunoprecipitated with TRAP1 in PC12 cells (Figure 1D), confirming that the identified PINK1–TRAP1 interaction occurs in vivo. We then assessed the interaction of TRAP1 with PD-linked PINK1 mutants (G309D, W437X, and L347P) and with putative catalytically inactive PINK1 mutants (K219A, D362A, D384A, and the triple mutant K219A/D362A/D384A [KDD]). None of the PINK1 mutations examined had any significant effect on the ability of PINK1 to bind TRAP1 (Figure 1E).

### PINK1 Colocalizes with TRAP1 in the Mitochondria

Next, we analyzed the intracellular distribution and colocalization of PINK1 and TRAP1 by using immunofluorescence confocal microscopy. PINK1 and TRAP1 both contain a predicted mitochondria targeting signal at their N-terminus, and a mitochondrial localization has previously been reported for PINK1 [12] as well as for TRAP1 [23]. In agreement with the earlier studies, we found that both PINK1 and TRAP1 proteins are localized to the mitochondria labeled by MitoTracker (Figure S1). Moreover, double labeling immunocytochemistry analysis revealed a substantial overlap between PINK1 and TRAP1 staining (Figure S1), suggesting that these two proteins colocalize in the mitochondria.

**Figure 2:**
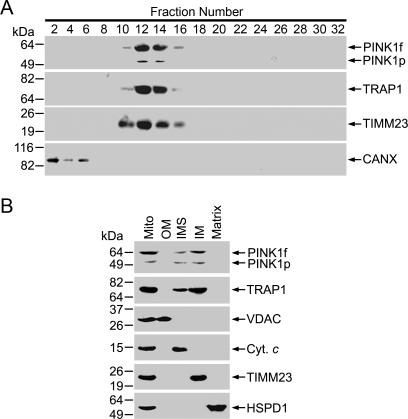
PINK1 and TRAP1 Colocalize in the Mitochondrial Inner Membrane and the Intermembrane Space (A) Post-nuclear supernatants of PC12 cells were fractionated on a 5%–15% linear Optiprep gradient, and the fractions were analyzed by immunoblotting for PINK1, TRAP1, TIMM23, and CANX. (B) Mitochondria (Mito) isolated from PC12 cells were fractionated into matrix, inner mitochondrial membrane (IM), intermembrane space (IMS), and outer mitochondrial membrane (OM) fractions, and analyzed by immunoblotting for PINK1, TRAP1, and markers of mitochondrial subcompartments: HSPD1 (matrix), TIMM23 (IM), cytochrome c (IMS), and VDAC (OM).

To complement our immunocytochemistry data, we used subcellular fractionation to characterize the intracellular distribution of PINK1 and TRAP1. Western blot analysis showed a clear co-fractionation of endogenous PINK1 and TRAP1 on a 5%–15% linear Optiprep gradient with mitochondria marker TIMM23, but not with endoplasmic reticulum marker CANX (Figure 2A). Further fractionation studies of the isolated mitochondria revealed that endogenous PINK1 and TRAP1 were both primarily found in the mitochondrial inner membrane and intermembrane space fractions (Figure 2B). Together, these results provide strong support for an in vivo association of PINK1 with TRAP1 in the mitochondria. Similar analyses of PC12 cells expressing wild-type PINK1 or PD-linked G309D, W437X, and L347P mutant PINK1 showed that the mitochondrial colocalization of PINK1 with TRAP1 was not affected by the pathogenic PINK1 mutations (Figure S2).

### PINK1 Phosphorylates TRAP1, and the Kinase Activity Is Abrogated by PD-linked Mutations

**Figure 3:**
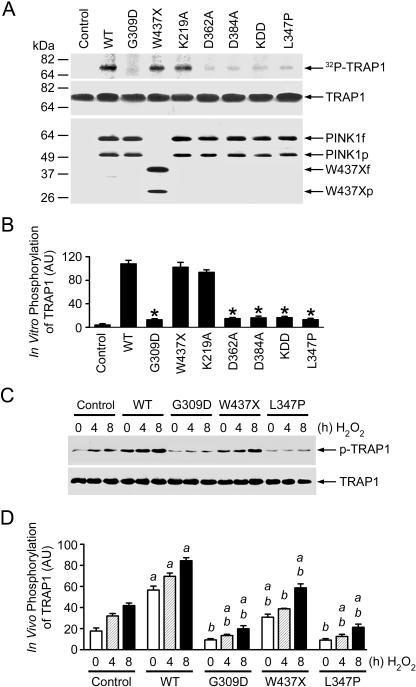
Phosphorylation of TRAP1 by Wild-Type and Mutant PINK1 (A) In vitro kinase assays were performed by incubation of purified TRAP1 with [γ^32^-P] ATP in the absence (control) or presence of FLAG-tagged wild-type (WT) or mutant PINK1 proteins as indicated. Phosphorylated TRAP1 was visualized by autoradiography (top panel). PINK1 and TRAP1 proteins used in the kinase assays were shown by immunoblotting with anti-TRAP1 (middle panel) and anti-FLAG antibodies (bottom panel). (B) Normalized levels of in vitro TRAP1 phosphorylation by wild-type or mutant PINK1. Data represent mean ± standard error of the mean (SEM) from three independent experiments. ^*^Significantly different from the wild-type PINK1 (*p* < 0.01). (C) PC12 cells expressing wild-type or mutant PINK1 or vector-transfected controls were treated with 400 μM H_2_O_2_ as indicated. In vivo phosphorylation of endogenous TRAP1 was determined by immunoprecipitation with anti-TRAP1 antibody followed by immunoblotting using anti-phosphoserine (upper panel) and anti-TRAP1 (lower panel) antibodies. (D) Normalized levels of in vivo TRAP1 phosphorylation by wild-type or mutant PINK1. Data represent mean ± SEM from three independent experiments. ^a^Significantly different from the corresponding H_2_O_2_-treated vector-transfected controls (*p* < 0.01). ^b^Significantly different from the wild-type PINK1-transfected cells (*p* < 0.01). AU, arbitrary units.

Our finding of a specific interaction and colocalization between TRAP1 and PINK1 raises the possibility that TRAP1 may be a substrate of PINK1 kinase. To examine this possibility, we performed in vitro kinase assays to investigate the ability of wild-type and mutant PINK1 to phosphorylate TRAP1. We observed a robust phosphorylation of TRAP1 by wild-type PINK1, as shown by the PINK1-mediated transfer of ^32^P from [γ-^32^P] ATP to the TRAP1 protein (Figure 3A). Computer modeling analysis of the PINK1 kinase domain suggests that the amino acid residues K219, D362, and D384 might be key determinants of PINK1 catalytic activity [11]. To test this prediction, we assessed the effects of single or triple mutations of these residues to alanines (K219A, D362A, D384A, or KDD) on the kinase activity of PINK1 in phosphorylating TRAP1. Although K219A mutation had no significant effect on the PINK1 kinase activity, the D362A, D384A, and KDD triple mutations dramatically reduced PINK1 activity in phosphorylating TRAP1 (Figure 3A and 3B). Analysis of the effects of PD-linked PINK1 mutations on the in vitro phosphorylation of TRAP1 revealed that the pathogenic mutation G309D or L347P virtually abolished the kinase activity of PINK1. In contrast, the kinase activity of PINK1 was not significantly affected by the W437X mutation (Figure 3A and 3B).

We next examined the effects of overexpressing wild-type or mutant PINK1 on the in vivo phosphorylation of endogenous TRAP1 in PC12 cells. As shown in Figure 3C, we observed a low basal level of TRAP1 phosphorylation in untransfected PC12 cells. TRAP1 phosphorylation was significantly increased in response to oxidative stress induced by H_2_O_2_. Overexpression of wild-type PINK1 resulted in a 3.3-fold increase in the basal level of TRAP1 phosphorylation and a 2.2-fold increase in oxidative-stress-induced TRAP1 phosphorylation (Figure 3C and 3D). The ability of PINK1 to promote in vivo TRAP1 phosphorylation was abolished by the PD-linked PINK1 mutations G309D and L347P. In fact, cells overexpressing G309D or L347P mutant PINK1 had approximately 50% lower levels of both basal and oxidative-stress-induced TRAP1 phosphorylation than the untransfected cells (Figure 3C and 3D). Since G309D and L347P mutations did not alter the interaction or colocalization of PINK1 with TRAP1 (Figures 1 and S2), our results suggest that these pathogenic PINK1 mutants have a dominant negative effect on TRAP1 phosphorylation in vivo by competing with endogenous PINK1 for binding of its substrate TRAP1. Interestingly, although the W437X mutation had no significant effect on PINK1-mediated TRAP1 phosphorylation in vitro (Figure 3A and 3B), the mutation caused a 30% to 45% reduction in both basal and oxidative-stress-induced levels of TRAP1 phosphorylation in vivo compared with wild-type PINK1 (Figure 3C and 3D). These results suggest that the W437X mutation has an inhibitory effect on the kinase activity of PINK1 in phosphorylating TRAP1 in vivo.

### PINK1 Protects against Oxidative-Stress-Induced Cell Death, and the Protective Action Depends on Its Kinase Activity

**Figure 4:**
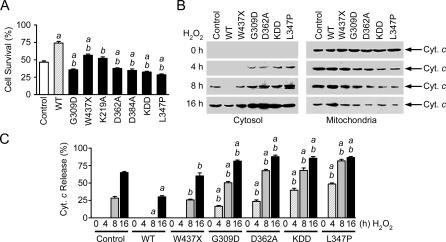
Wild-Type PINK1, but Not Kinase-Dead or PD-Linked Mutant PINK1, Protects against Oxidative-Stress-Induced Apoptosis (A) PC12 cells expressing wild-type (WT) or mutant PINK1 or vector-transfected controls were treated with 400 μM H_2_O_2_ for 16 h. The extent of cell survival was assessed by using the MTT assay. Data represent mean ± SEM from three independent experiments. ^a^Significantly different from the vector-transfected controls (*p* < 0.01). ^b^Significantly different from the wild-type PINK1-transfected cells (*p* < 0.01). (B) Immunoblot analysis of cytochrome c (Cyt. c) in the cytosol and mitochondria fractions isolated from PC12 cells expressing wild-type or mutant PINK1 after treatment with 400 μM H_2_O_2_ for the indicated times. (C) The level of cytochrome c released to the cytosol is normalized to the total level of cytochrome c in each cell sample. Data represent mean ± SEM from three independent experiments. ^a^Significantly different from the corresponding H_2_O_2_-treated vector-transfected controls (*p* < 0.01). ^b^Significantly different from the wild-type PINK1-transfected cells (*p* < 0.01).

Given the observed effects of PINK1 on oxidative-stress-induced phosphorylation of TRAP1 (Figure 3), we investigated the roles of wild-type and mutant PINK1 proteins in cell vulnerability to oxidative stress. PC12 cells were transfected with C-terminally FLAG-tagged wild-type or mutant PINK1 to achieve similar levels of exogenous PINK1 protein expression, as confirmed by Western blot analysis (data not shown). Cell viability assays revealed that treatment of untransfected or vector-transfected PC12 cells with H_2_O_2_ resulted in cell death (Figure 4A; and additional data not shown). Cells expressing exogenous wild-type PINK1 were much more resistant to H_2_O_2_-induced cell death (Figure 4A), supporting a protective role of PINK1 against oxidative stress. The protective effect of PINK1 was significantly decreased by the putative kinase-dead K219A mutation and was completely abolished by the catalytically inactive D362A, D384A, and KDD triple mutations (Figure 4A), indicating that the cytoprotective function of PINK1 depends on its kinase activity.

Analysis of the effects of PD-linked PINK1 mutations on cell vulnerability revealed that the pathogenic W437X mutation significantly reduced the ability of PINK1 to protect against oxidative-stress-induced cell death (Figure 4A). We found that the pathogenic G309D and L347P mutations not only abolished the cytoprotective effect of PINK1, but also exacerbated oxidative-stress-induced cell death (Figure 4A), suggesting a dominant negative effect of these mutations on cell survival.

### PINK1 Prevents Oxidative-Stress-Induced Cell Death by Suppressing Cytochrome c Release from Mitochondria

The observed mitochondrial localization (Figure 2) and cytoprotective action (Figure 4A) of PINK1 raise the possibility that PINK1 may regulate the mitochondrial cell death pathway. To test this possibility, we examined the effects of overexpressing wild-type and mutant PINK1 proteins on the release of cytochrome c from mitochondria, a crucial step in the mitochondrial apoptotic cascade. As shown in Figure 4B, treatment of vector-transfected PC12 cells with H_2_O_2_ induced cytochrome c release from mitochondria to the cytosol in a time-dependent manner. Overexpression of wild-type PINK1 caused a significant reduction in the release of cytochrome c under the same oxidative stress conditions (Figure 4B and 4C). The inhibitory effect of PINK1 on cytochrome c release was completely abrogated by the catalytically inactive D362A mutation and the KDD triple mutations (Figure 4B and 4C), suggesting the critical involvement of PINK1 kinase activity in regulation of mitochondrial apoptotic pathway.

Analysis of the effects of PD-linked PINK1 mutations on cytochrome c release showed that the W437X mutation caused a significant increase in H_2_O_2_-induced cytochrome c release compared to cells expressing similar level of exogenous wild-type PINK1 (Figure 4B and 4C), in agreement with the observed effect of the W437X mutation on the cytoprotective function of PINK1 (Figure 4A). The pathogenic G309D and L347P mutations not only abolished the ability of PINK1 to suppress cytochrome c release, but also accelerated H_2_O_2_-induced cytochrome c release (Figure 4B and 4C), consistent with the dominant negative effects of these mutations on cell survival (Figure 4A). Western blot analysis confirmed that the relative distribution of PINK1 in the mitochondria and cytosol fractions was not altered by the H_2_O_2_ treatment or by the PINK1 mutations examined (data not shown), suggesting that the observed effects of wild-type and mutant PINK1 proteins on cell survival are mediated through their action in mitochondria on the release of cytochrome c rather than by altering the subcellular localization of PINK1.

### PINK1 Is Essential for Phosphorylation of TRAP1 and Protection against Oxidative-Stress-Induced Apoptosis

**Figure 5:**
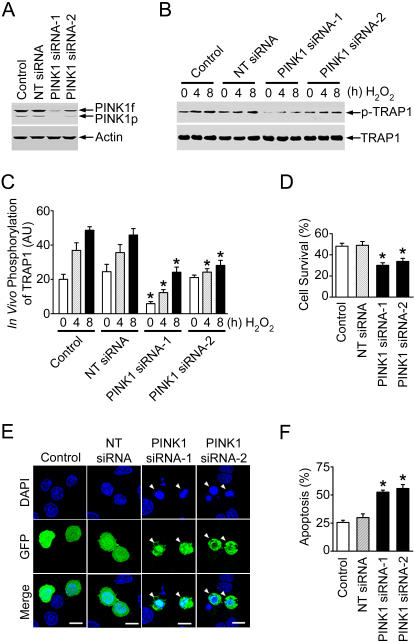
PINK1 Knockdown Reduces TRAP1 Phosphorylation and Protection against Oxidative Stress (A) PC12 cells were transfected with vehicle (control), NT siRNA, or PINK1-specific siRNAs (PINK1 siRNA-1 and PINK1 siRNA-2). The levels of PINK1 and actin in the cell lysates were analyzed by immunoblotting with anti-PINK1 and anti-actin antibodies. (B) PC12 cells transfected with the indicated siRNA or vehicle (control) were treated with 400 μM H_2_O_2_ for the indicated times. In vivo phosphorylation of endogenous TRAP1 was determined by immunoprecipitation with anti-TRAP1 antibody followed by immunoblotting using anti-phosphoserine and anti-TRAP1 antibodies. (C) Normalized levels of in vivo TRAP1 phosphorylation in the control and siRNA-transfected cells. Data represent mean ± SEM from three independent experiments. ^*^Significantly different from the corresponding H_2_O_2_-treated control cells (*p* < 0.01). AU, arbitrary units. (D) PC12 cells transfected with vehicle (control) or the indicated siRNAs were treated with 400 μM H_2_O_2_ for 16 h. Cell viability was assessed by using the MTT assay. Data represent mean ± SEM from three independent experiments. ^*^Significantly different from the H_2_O_2_-treated control cells (*p* < 0.01). (E) PC12 cells co-transfected with an expression vector encoding enhanced green fluorescent protein (pEGFP) and vehicle (control) or indicated siRNAs were treated with 400 μM H_2_O_2_ for 16 h. Transfected cells were shown by the green fluorescence emitted by green fluorescent protein (GFP), and nuclear morphology was visualized by DAPI staining (blue). Arrowheads indicate transfected cells with apoptotic nuclei. Scale bar, 10 μm. (F) Apoptosis is expressed as the percentage of transfected cells with apoptotic nuclear morphology. Data represent mean ± SEM from three independent experiments. ^*^Significantly different from the H_2_O_2_-treated control cells (*p* < 0.01).

To provide further evidence supporting the role of PINK1 in regulation of TRAP1 phosphorylation and mitochondrial apoptotic signaling, we examined the effect of siRNA-mediated PINK1 knockdown on TRAP1 phosphorylation and oxidative-stress-induced apoptosis. As shown in Figure 5A, PINK1 siRNA-1 and PINK1 siRNA-2, two distinct siRNA duplexes targeting different regions of PINK1 mRNA, both specifically inhibited the expression of endogenous PINK1 in PC12 cells. We found that, in cells transfected with either PINK1 siRNA-1 or PINK1 siRNA-2, H_2_O_2_-induced phosphorylation of endogenous TRAP1 was significantly decreased compared with the cells transfected with non-targeting (NT) control siRNA or with vehicle alone (Figure 5B and 5C). These results further confirm that TRAP1 is indeed a substrate of PINK1 kinase.

**Figure 6:**
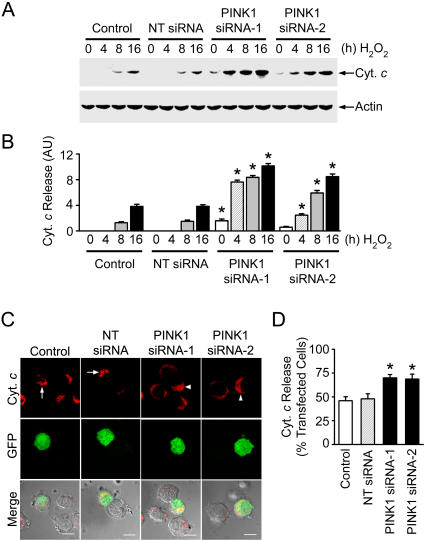
PINK1 Depletion Increases Cytochrome c Release from Mitochondria (A) PC12 cells transfected with the indicated siRNA or vehicle (control) were treated with 400 μM H_2_O_2_ for the indicated times. The levels of cytochrome c (Cyt. c) and actin in the cytosol were determined by immunoblotting with anti-cytochrome c and anti-actin antibodies. (B) The level of cytochrome c released to the cytosol is normalized to the level of actin in each cell sample. Data represent mean ± SEM from three independent experiments. ^*^Significantly different from the corresponding H_2_O_2_-treated control cells (*p* < 0.01). AU, arbitrary units. (C) PC12 cells co-transfected with pEGFP and vehicle (control) or indicated siRNAs were treated with 400 μM H_2_O_2_ for 16 h. Cell morphology was imaged by using phase-contrast microscopy (grey), transfected cells were visualized by the green fluorescence emitted by green fluorescent protein (GFP), and the cellular distribution of cytochrome c was detected by immunostaining with anti-cytochrome c antibody (red). Transfected cells with mitochondrial cytochrome c staining are indicated by arrows, and those with diffuse, cytosolic cytochrome c staining are indicated by arrowheads. Scale bar, 10 μm. (D) Quantification of the percentage of transfected cells showing cytochrome c release. Data represent mean ± SEM from three independent experiments. ^*^Significantly different from the H_2_O_2_-treated control cells (*p* < 0.05).

Cell viability and nuclear morphology analyses revealed that depletion of endogenous PINK1 by PINK1 siRNA-1 or PINK1 siRNA-2 enhanced the susceptibility of PC12 cells to oxidative-stress-induced apoptosis (Figure 5D–5F). Moreover, PINK1 depletion significantly increased oxidative-stress-induced release of cytochrome c from mitochondria to the cytosol (Figure 6). Together, these data provide strong support for a critical role of PINK1 in regulation of the mitochondrial apoptotic pathway.

### TRAP1 Is Required for PINK1-Mediated Protection against Oxidative-Stress-Induced Apoptosis

**Figure 7:**
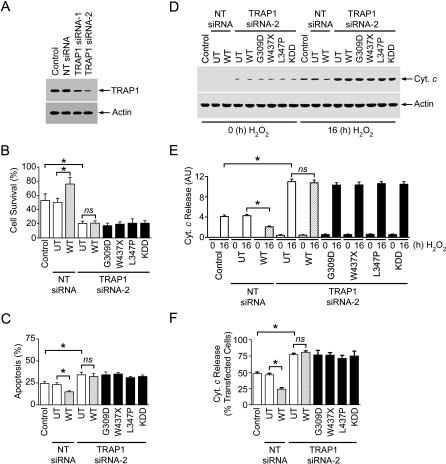
TRAP1 Depletion Abolishes the Effects of Wild-Type and Mutant PINK1 on Cell Vulnerability to Oxidative Stress (A) PC12 cells were transfected with vehicle (control), NT siRNA, or TRAP1-specific siRNAs (TRAP1 siRNA-1 and TRAP1 siRNA-2). The levels of TRAP1 and actin in the cell lysates were analyzed by immunoblotting with anti-TRAP1 and anti-actin antibodies. (B) PC12 cells treated with TRAP1 siRNA-2 or NT siRNA were either untransfected (UT) or transfected with wild-type (WT) or mutant PINK1 as indicated. Vehicle-treated, non-transfected PC12 cells were used as the control. Cells were exposed to 400 μM H_2_O_2_ for 16 h, and the extent of cell survival was assessed by using the MTT assay. Data represent mean ± SEM from three independent experiments. *, *p* < 0.05; ns, not significant. (C) PC12 cells co-transfected with pEGFP and vehicle (control) or indicated siRNAs and PINK1 plasmids were treated with 400 μM H_2_O_2_ for 16 h, and the extent of apoptosis was determined by morphological analysis of DAPI-stained nuclei. The percentage of transfected cells with apoptotic nuclear morphology was quantified. Data represent mean ± SEM from three independent experiments. ^*^, *p* < 0.05; ns, not significant. (D) The levels of cytochrome c (Cyt. c) and actin in the cytosol fractions from cells described in (B) were determined by immunoblotting with anti-cytochrome c and anti-actin antibodies. (E) The level of cytochrome c released to the cytosol is normalized to the level of actin in each cell sample. Data represent mean ± SEM from three independent experiments. ^*^, *p* < 0.01; ns, not significant. AU, arbitrary units. (F) PC12 cells co-transfected with pEGFP and vehicle (control) or indicated siRNAs and PINK1 plasmids were treated with 400 μM H_2_O_2_ for 16 h, and the cellular distribution of cytochrome c was detected by immunostaining with anti-cytochrome c antibody. The percentage of transfected cells showing cytochrome c release was quantified. Data represent mean ± SEM from three independent experiments. ^*^, *p* < 0.05; ns, not significant.

Our finding that TRAP1 phosphorylation by PINK1 correlates with the anti-apoptotic activity of PINK1 raises the possibility that TRAP1 may be the intermediate between PINK1 and apoptosis inhibition. To test this possibility, we investigated the effect of siRNA-mediated TRAP1 knockdown on the cytoprotective function of PINK1. Two different TRAP1-targeting siRNAs (TRAP1 siRNA-1 and TRAP1 siRNA-2) were used to reduce the expression of endogenous TRAP1 in PC12 cells. Western blot analysis showed that TRAP1 siRNA-2 caused a greater decrease in TRAP1 protein level than TRAP1 siRNA-1, whereas the NT control siRNA had no effect on TRAP1 expression (Figure 7A). Analyses of cell viability, nuclear morphology, and cytochrome c release revealed that TRAP1 depletion resulted in a significant increase in oxidative-stress-induced cytochrome c release and cell death (Figure 7B–7F). We found that, in contrast to the protective effect of overexpressing wild-type PINK1 seen in the control (Figure 4) or in the NT siRNA-treated PC12 cells (Figure 7), overexpression of wild-type PINK1 in TRAP1-depleted PC12 cells was no longer able to inhibit oxidative-stress-induced cytochrome c release and cell death (Figure 7B–7F). Moreover, the dominant negative effects on cell survival observed with PD-linked G309D and L347P mutant PINK1 (Figure 4) were also abolished by TRAP1 depletion (Figure 7B–7F). Taken together, these results provide evidence supporting a role of TRAP1 as the downstream effector of PINK1 in protection against oxidative-stress-induced apoptosis.

Given our result that TRAP1 is localized in the mitochondrial inner membrane and the intermembrane space where cytochrome c is located (Figure 2B), we next investigated whether TRAP1 may exert its anti-apoptotic function by binding directly to cytochrome c, thereby preventing it from being released from mitochondria. Co-immunoprecipitation experiments failed to find TRAP1 in physical association with cytochrome c either in the absence or presence of wild-type or mutant PINK1 or under conditions of oxidative stress (Figure S3), arguing against the possibility that TRAP1-mediated inhibition of cytochrome c release occurs through a direct interaction/retention mechanism.

## Discussion

Although mutations in the putative kinase PINK1 are a common cause of autosomal recessive PD, very little is known about the biological function of PINK1 and how loss of PINK1 function leads to neurodegeneration. The present study demonstrates that PINK1 is a mitochondrial serine/threonine kinase with a critical role in promoting cell survival. We have identified what is to our knowledge the first substrate for the PINK1 kinase: the mitochondrial molecular chaperone TRAP1. Our findings reveal a hitherto unexplored pathway by which PINK1 phosphorylates downstream effector TRAP1 to protect cells against apoptosis, and suggest a link between the impairment of this novel mitochondrial pathway and neurodegeneration in PD.

It has become increasingly clear that molecular chaperones not only facilitate protein folding, but also regulate a number of cellular processes, including cell survival and apoptosis [24,25]. Despite the critical importance of mitochondria in cell function and survival, the current knowledge about mitochondrial molecular chaperones is limited. TRAP1 is a poorly characterized mitochondrial protein with significant sequence homology to the HSP90AA1 family of molecular chaperones [23]. Our finding that siRNA-mediated depletion of TRAP1 sensitizes cells to oxidative-stress-induced cytochrome c release and cell death suggests a role for TRAP1 in the modulation of the mitochondrial apoptotic cascade. Consistent with this role, TRAP1 knockdown has been shown to enhance cytochrome c release and apoptosis induced by other stressors, such as protein-tyrosine kinase inhibitor β-hydroxyisovalerylshikonin and topoisomerase II inhibitor VP16 [26]. The molecular basis of the anti-apoptotic function of TRAP1 remains unknown. Although we find that TRAP1 and cytochrome c colocalize in the mitochondrial intermembrane space, our results indicate that TRAP1 does not physically associate with cytochrome c in cells either in the absence or presence of oxidative stress. Thus, TRAP1 may inhibit the release of cytochrome c from mitochondria through a mechanism other than direct interaction/retention. Future studies are required to elucidate the mechanism by which TRAP1 regulates cytochrome c release and apoptosis.

Our data reveal that endogenous TRAP1 is constitutively phosphorylated at low levels, and the TRAP1 phosphorylation is significantly increased in response to oxidative stress induced by H_2_O_2_. Both constitutive and oxidative-stress-induced TRAP1 phosphorylation are dramatically enhanced by PINK1 overexpression and are reduced by siRNA-mediated depletion of PINK1, indicating that PINK1 is a major kinase responsible for phosphorylating TRAP1 in cells. These results, together with the in vitro phosphorylation data, provide compelling evidence that TRAP1 is a bona fide substrate for PINK1 kinase. We find that overexpression of PINK1 protects cells against oxidative-stress-induced apoptosis by suppressing cytochrome c release from mitochondria, and this protective action of PINK1 depends on its kinase activity to phosphorylate TRAP1. Conversely, PINK1 depletion causes increased cytochrome c release from mitochondria and sensitizes cells to oxidative-stress-induced cell death, and the pro-apoptotic effect of PINK1 depletion correlates with reduced TRAP1 phosphorylation. Moreover, our studies using TRAP1 siRNAs have provided direct evidence that TRAP1 is required for PINK1-mediated protection against oxidative-stress-induced cytochrome c release and cell death. Collectively, these findings point to a specific pathway in which PINK1 exerts its cytoprotective function by phosphorylating downstream effector TRAP1 to inhibit the release of cytochrome c from mitochondria.

Our work indicates that the mitochondrial PINK1–TRAP1 anti-apoptotic pathway is disrupted by PD-linked PINK1 G309D, L347P, and W437X mutations. Previous studies of PINK1 mutations using in vitro autophosphorylation assays and artificial kinase substrates have led to conflicting results. For example, analyses with recombinant GST-fused PINK1 proteins purified from Escherichia coli showed that PINK1 autophosphorylation activity was slightly decreased by the G309D mutation [11,12] and was significantly increased by the W437X mutation [12] and that neither of these mutations affected the activity of PINK1 to phosphorylate artificial substrate α-casein [12]. In contrast, a recent study using recombinant PINK1 proteins isolated from baculovirus-infected *sf9* insect cells failed to detect PINK1 autophosphorylation activity, but showed that PINK1 C-terminal truncation reduced the activity of PINK1 to phosphorylate artificial substrate histone H1 [27]. These discrepancies underscore the importance of knowing physiological substrate(s) for characterizing the kinase function of wild-type and mutant PINK1. Our analyses reveal that single G309D and L347P mutations abolish the kinase activity of PINK1 to phosphorylate its physiological substrate TRAP1 both in vitro and in vivo. By comparison, the W437X mutation has a weaker effect on PINK1-mediated TRAP1 phosphorylation. Our findings that PD-linked mutations impair the ability of PINK1 to promote TRAP1 phosphorylation and cell survival provide strong support for a loss-of-function pathogenic mechanism in familial PD patients carrying homozygous PINK1 mutations.

Recently, an increasing number of PD cases with heterozygous mutations affecting only one *PINK1* allele have been reported, including single heterozygous G309D and W437X mutations [6–10]. Our results that PD-linked PINK1 mutations specifically inhibit PINK1 kinase function without affecting the substrate binding or subcellular localization of PINK1 raise an interesting possibility that these mutants may act in a dominant negative manner to inhibit the function of the normal *PINK1* allele by competing with endogenous wild-type PINK1 for binding its substrate TRAP1. In support of this possibility, we find that PD-linked PINK1 G309D and L347P mutations have a dominant negative effect on in vivo TRAP1 phosphorylation and cell survival. These results suggest a dominant negative pathogenic mechanism by which heterozygous PINK1 mutations lead to Parkinsonian symptoms [6,7,9]. By comparison, overexpression studies show that the W437X mutation has a smaller but significant inhibitory effect on in vivo TRAP1 phosphorylation and cell survival, providing evidence supporting a pathogenic role of the heterozygous W437X mutation. Our finding of a mitochondrial PINK1–TRAP1 anti-apoptotic pathway and its impairment by PD-linked mutations provides new insights into the pathogenic mechanisms of PD and suggests novel targets for therapeutic intervention.

## Materials and Methods

### Plasmids and anti-PINK1 antibody.

Conventional molecular biological techniques [28] were used to generate the C-terminally FLAG-tagged human PINK1 (PINK1-FLAG), the PD-linked G309D, W437X, and L347P mutant PINK1, and the putative catalytically inactive PINK1 mutants, K219A, D362A, D384A, and the KDD triple mutant. A polyclonal anti-PINK1 antibody was raised in rabbit against amino acids 135–155 of human PINK1, and affinity purified as described previously [29].

### Affinity purification and mass spectrometry.

HeLa cells transfected with pPINK1-FLAG or the vector control were lysed in a buffer containing 50 mM Tris-HCl (pH 7.4), 150 mM NaCl, 1 mM EDTA, 1% Triton X-100, and protease inhibitor cocktail (Sigma, http://www.sigmaaldrich.com). The lysates were affinity purified with anti-FLAG M2 affinity gel (Sigma). FLAG-tagged PINK1-protein complexes were eluted into 1-ml fractions and analyzed by immunoblotting with anti-FLAG antibody (Roche Applied Science, http://www.roche-applied-science.com). The fractions containing FLAG-tagged PINK1 protein were pooled and resolved on SDS-PAGE. The proteins were then transferred to PVDF membrane followed by Ponceau S staining. Protein bands were excised from the membrane, digested by trypsin, and analyzed by nanoelectrospray tandem mass spectrometry as described previously [30,31].

### Cell transfections and immunoprecipitations.

HeLa and PC12 cells were transfected with the indicated plasmids by using Lipofectamine 2000 (Invitrogen, http://www.invitrogen.com) and TransIT neuronal transfection reagent (Mirus, http://www.mirusbio.com), respectively, according to the manufacturer's instructions. Immunoprecipitations were performed at 24 h post-transfection as described [32] using the indicated antibodies. The immunocomplexes were resolved by SDS-PAGE and analyzed by immunoblotting [32].

### In vitro and in vivo phosphorylation assays.

In vitro kinase assays were performed as described [33] by incubation of affinity-purified FLAG-tagged wild-type or mutant PINK1 with TRAP1 for 20 min at 30 °C in 100 μl of reaction buffer containing 50 mM Tris-HCl (pH 7.5), 8 mM MgCl_2_, 2 mM MnCl_2_, 0.1 mM ATP, and 5 μCi of [γ-^32^P] ATP. The reaction mixtures were resolved by SDS-PAGE, and phosphorylated TRAP1 was visualized by autoradiography. The relative level of in vitro TRAP1 phosphorylation was measured by quantifying the amount of ^32^P incorporated into TRAP1 and was normalized against the level of total TRAP1. For detection of in vivo phosphorylation of TRAP1, endogenous TRAP1 protein was immunoprecipitated by using anti-TRAP1 antibody (BD Biosciences, http://www.bdbiosciences.com), followed by immunoblotting with anti-phosphoserine antibody (Invitrogen). The relative level of in vivo TRAP1 phosphorylation was determined by measuring the intensity of the phosphoserine-immunoreactive band and was normalized against the level of total TRAP1.

### Immunofluorescence confocal microscopy.

Double labeling indirect immunofluorescence microscopy was performed as described previously [29,34,35]. Mitochondria were labeled with MitoTracker (Molecular Probes, http://probes.invitrogen.com) prior to fixation. Cells were then immunostained with the indicated antibodies, and examined by using a Zeiss (http://www.zeiss.com) LSM 510 confocal microscope.

### Subcellular fractionations.

For separation of mitochondria and other organelles, PC12 cells were homogenized and fractionated on a 5%–15% linear Optiprep (Nycomed, http://www.nycomed.com) gradient as described previously [32]. For sub-mitochondria fractionation, mitochondria isolated from PC12 cells were further separated by differential centrifugation into the matrix, the inner mitochondrial membrane, the intermembrane space, and the outer mitochondrial membrane fractions as described [36].

### siRNA transfection.

For depletion of PINK1 or TRAP1 in PC12 cells, siRNAs (Dharmacon, http://www.dharmacon.com) were generated against the following rat PINK1 or TRAP1 mRNA sequences: PINK1 siRNA-1, 5′-CAAGUCCGACAACAUACUU-3′; PINK1 siRNA-2, 5′-GCGUGUUGGCCUGCAGUUA-3′; TRAP1 siRNA-1, 5′-GCAAAGAUUUCGCCAACGA-3′; and TRAP1 siRNA-2, 5′-GAACAUUCACCCUAUUAUG-3′. A control siRNA with no known mammalian homology (siCONTROL Non-Targeting siRNA #1, Dharmacon) was used as negative control. PC12 cells were transfected with the indicated siRNAs using the TransIT siQUEST (Mirus) reagent as described previously [37].

### Cell viability and apoptosis assays.

PC12 cells were treated with H_2_O_2_ as indicated, and cell viability was assessed using the 3-(4,5-dimethylthiazol-2-yl)-2,5-diphenyltetrazolium bromide (MTT) assay as described [38]. Apoptotic cell death was measured by morphological assessment of nuclei stained with 4′,6-diamidino-2-phenylindole (DAPI, Molecular Probes) as previously described [39]. The percentage of transfected cells with nuclear shrinkage and chromatin condensation was scored for apoptosis in a blinded manner.

### Cytochrome c release assays.

Biochemical assays of cytochrome c release were performed as described [40]. Briefly, PC12 cells were homogenized and then fractionated into the mitochondria and cytosol fractions. The release of cytochrome c from mitochondria to the cytosol was determined by immunoblotting with anti-cytochrome c antibody (clone 7H8.2C12, BD Biosciences). For cell-based assays of cytochrome c release, the cellular distribution of cytochrome c was determined by immunofluorescence confocal microscopy using anti-cytochrome c antibody (clone 6H2.B4, BD Biosciences) as described [41]. The release of cytochrome c from mitochondria to the cytosol was indicated by a transition from a punctate to a diffuse cytochrome c immunostaining pattern. The percentage of transfected cells showing cytochrome c release was scored in a blinded manner.

### Statistical analysis.

Statistical analysis of the data was performed by using ANOVA with Tukey's post hoc analysis.

## Supporting Information

Figure S1 Colocalization of PINK1 with TRAP1 in the Mitochondria Untransfected HeLa cells (D–F) or transfected HeLa cells expressing PINK1-FLAG (A–C) or PINK1-HA (G–I) were analyzed by double labeling confocal microscopy with MitoTracker (B and E), anti-FLAG (A), anti-TRAP1 (D and H), and anti-HA (G) antibodies. Scale bar, 10 μm.
(12 MB TIF)

Figure S2 PD-Associated PINK1 Mutations Have No Effect on the Mitochondrial Colocalization of PINK1 with TRAP1 Mitochondria (Mito) isolated from transfected PC12 cells expressing wild-type PINK1 or PD-linked G309D, W437X, or L347P mutant PINK1 were fractionated into matrix, inner mitochondrial membrane (IM), intermembrane space (IMS), and outer mitochondrial membrane (OM) fractions, and analyzed by immunoblotting for PINK1 and TRAP1.
(1.6 MB TIF)

Figure S3 TRAP1 Does Not Associate with Cytochrome c in the Absence or Presence of PINK1 and Oxidative Stress (A) Lysates from untransfected (UT) or transfected PC12 cells expressing wild-type (WT) PINK1 or the indicated mutant PINK1 were immunoprecipitated with anti-TRAP1 antibody, followed by immunoblotting using antibodies against TRAP1 and cytochrome c (Cyt. c). “Input” lane shows the levels of endogenous TRAP1 and cytochrome c in the lysate of untransfected PC12 cells.
(B) PC12 cells expressing wild-type PINK1 or vector-transfected controls were incubated in the presence or absence of 400 μM H_2_O_2_ for 16 h. Cell lysates (Input) were subjected to immunoprecipitation with anti-TRAP1, followed by immunoblotting using antibodies against TRAP1 and cytochrome c (Cyt. c).
(1.2 MB TIF)

### Accession Numbers

The GenBank (http://www.ncbi.nlm.nih.gov/Genbank) accession number for TRAP1 is NP_057376.

## Abbreviations

DAPI: 4′,6-diamidino-2-phenylindole
KDD: K219A/D362A/D384A
MTT: 3-(4,5-dimethylthiazol-2-yl)-2,5-diphenyltetrazolium bromide
NT: non-targeting
PD: Parkinson disease
PINK1: PTEN induced putative kinase 1
SEM: standard error of the mean
siRNA: small interfering RNA
TRAP1: TNF receptor-associated protein 1
